# An Electric Freak

**Published:** 1891-08

**Authors:** J. W. Duncan

**Affiliations:** Atlanta, Ga.


### AN ELECTRIC FREAK.

BY J. W. DUNCAN, M. D., ATLANTA, GA.

Ella Davis, colored, age 13 years, was in the garden with her sister, June 8th, 2:30 p. m., at which time there was a severe electric and rain storm passing over the city. Ella was stricken down, while her sister was only slightly shocked. Ella was apparently dead for half hour or more. I was only one block from the place, and I was called at once. I told the father of the girl to run back and pour water on her until I got there. I failed to get the number of the house, and it was some half hour before I found the place; but they had followed my orders, and she was breathing irregularly when I got there. They said she did not breathe for half hour after she was stricken. I continued the free application of water until 7 p. m. She became rational about 11 p. m. This is the ninth day since the accident. Her pulse is 100, her temperature 103. Complains of nothing except weakness.

The strange points in this case are the lines or stripes. One begins over the mastoid process, on the right side, extending down the neck over the right scapula, down the side over right hip and thigh, and stops abruptly at popliteal space; another line starts at point of right shoulder, and passing one inch to the right of nipple, extends over chest and abdomen to umbilicus; and a third line begins at the top of the sternum and extends down the left side of the body and thigh to the popliteal space.

These lines are well marked, and the epidermis has peeled off very much like there had been a hot iron passed over the parts. They are from one to one and a-half inches in width. I am of the opinion that the immediate and free use of water saved her life thus far. I do not know what the termination will be. How a person can live after receiving a stroke sufficiently strong to blister the body, as it seems to have done in this case, I am unable to understand.

P. S. June 30th.—I have looked after the case of Ella Davis with some interest. This is the 22d day since she was injured. The temperature continued from 102 to 103 for several days. At this time it is only 99 deg. The pulse has continued about 100. I cannot say that the shock was the cause of the fever, but I believe it was the exciting cause. There was no intestinal trouble, as is common in typhoid fever. The tongue was only slightly furred. She is now out of danger, so far as I can see.

